# Supplementary material for: Large-Scale Transcriptome Profiling and Network Pharmacology Analysis Reveal the Multi-Target Inhibitory Mechanism of Modified Guizhi Fuling Decoction in Prostate Cancer Cells
Source: Pharmaceuticals (Basel). 2025 Aug 27;18(9):1275. doi: 10.3390/ph18091275 (PMC12472799; doi:10.3390/ph18091275)
Supplement: Supplementary file 1 [file pharmaceuticals-18-01275-s001.zip › English-Editing-Certificate.pdf]

We certify that the following article

Large-Scale Transcriptome Profiling and Network Pharmacology Analysis Reveal the Multi-Target Inhibitory Mechanism of Modified Guizhi Fuling Decoction in Prostate Cancer Cells

Guochen Zhang, Lei Xiang, Qingzhou Li, Mingming Wei, Xiankuo Yu, Yan Luo, Jianping Chen, Xilinqiqige Bao, Dong Wang *, Shiyi Zhou *

has undergone English language editing by MDPI. The text has been checked for correct use of grammar and common te[illegible] terms, and edited to a level suitable for reporting research in a scholarly journal.

MDPI uses experienced, native English speaking editors. Full details of the editing service can be found at ► https://www.mdpi.com/authors/english.

MDPI

Basel, Switzerland
August 2025

AUTHORservices

english-98939
