# Supplementary material for: Large-Scale Transcriptome Profiling and Network Pharmacology Analysis Reveal the Multi-Target Inhibitory Mechanism of Modified Guizhi Fuling Decoction in Prostate Cancer Cells
Source: Pharmaceuticals (Basel). 2025 Aug 27;18(9):1275. doi: 10.3390/ph18091275 (PMC12472799; doi:10.3390/ph18091275)
Supplement: Supplementary file 1 [file pharmaceuticals-18-01275-s001.zip › proofed supplementary figure.pdf]

Supplementary data

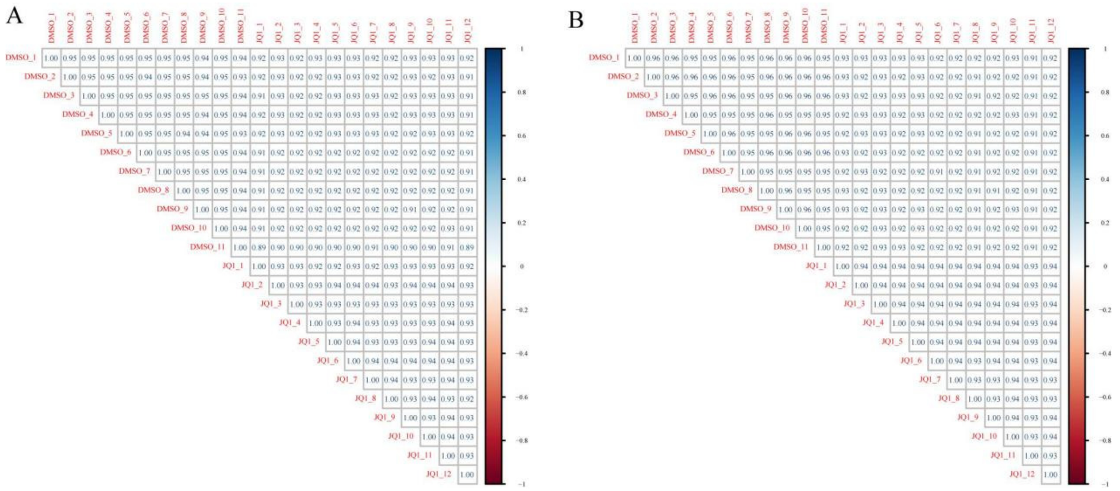

Figure S1. Results of Pearson correlation test. (A) The results in DU145 cells, (B) The results in PC3 cells.

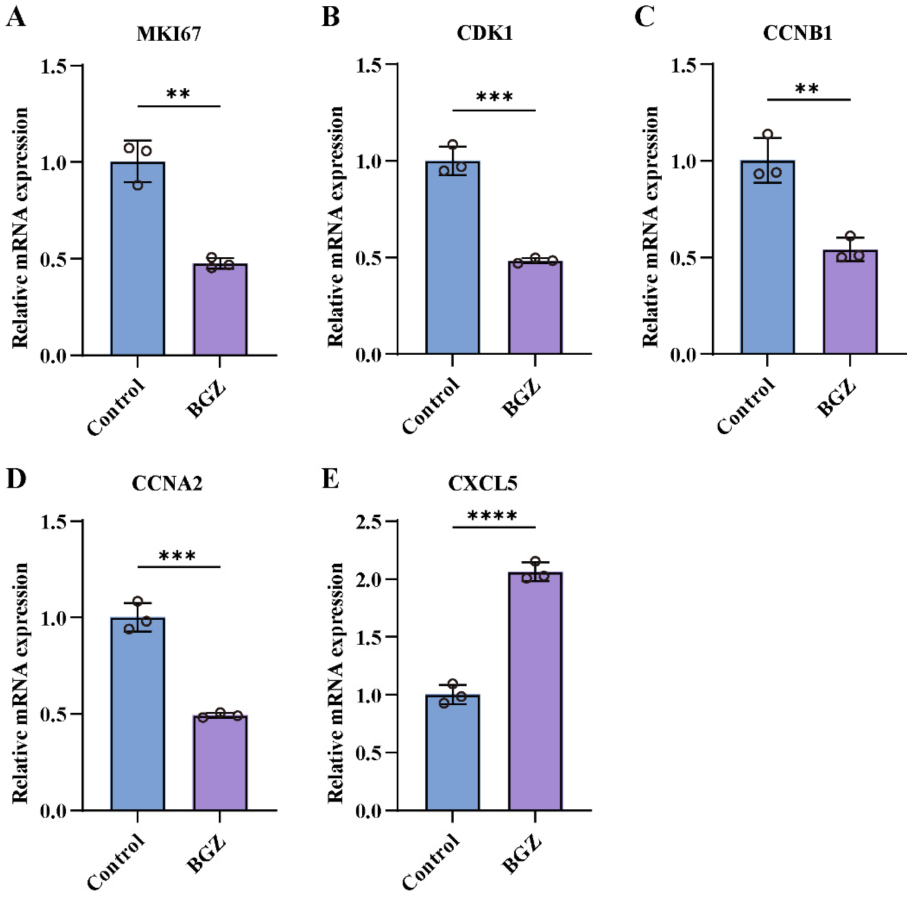

Figure S2. The mRNA expression levels of genes in DU145 cells were measured by qRT-PCR after treatment with Buguzhi (100 μg/mL). (A), MKI67, (B) CDK1, (C) CCNB1, (D) CCNA2, (E) CXCL5. (BGZ, Buguzhi). Data are presented as means ± SD, ****$P < 0.0001$, ***$P < 0.001$, **$P < 0.01$.

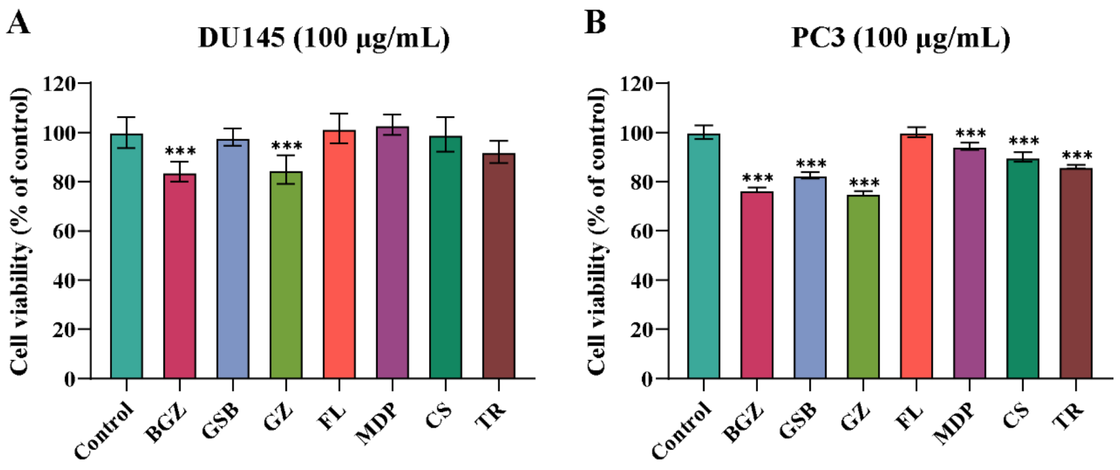

Figure S3. Cell viability of DU145 and PC3 cells treated with seven different herbal extracts (100 μg/mL) was determined by CCK8 assay. (A) The results in DU145 cells, (B) The results in PC3 cells. Data are presented as means ± SD, $***P < 0.001$ vs. control group. (FL, Fuling; MDP, Mudanpi; BGZ, Buguzhi; GZ, Guizhi; GSB, Gusuibu; CS, Chishao; TR, Taoren)

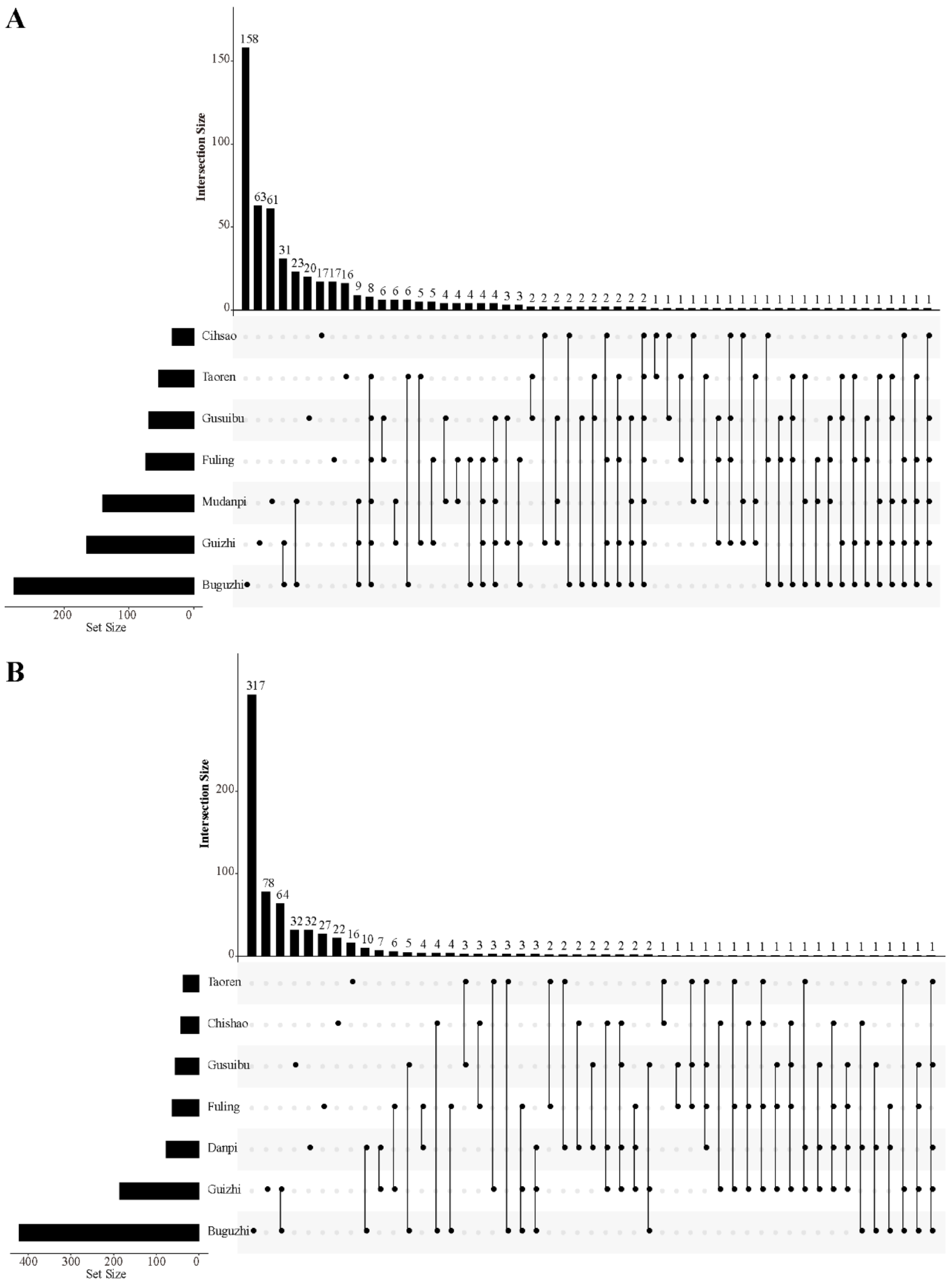

Figure S4. UpSet plots of the DEGs of the seven herbs across. (A) The results in DU145 cells, (B) The results in PC3 cells.

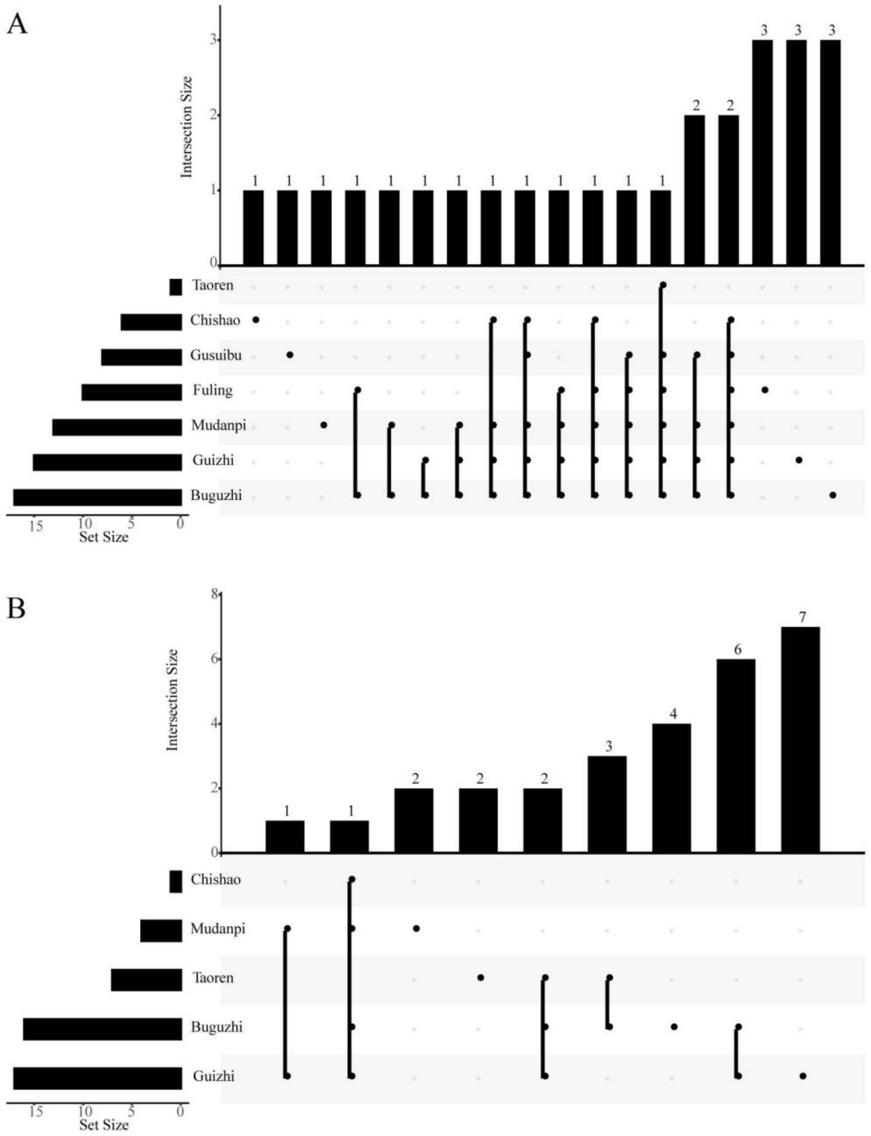

Figure S5. UpSet plots of the enriched gene sets of the seven herbs across. (A) The results in DU145 cells, (B) The results in PC3 cells.

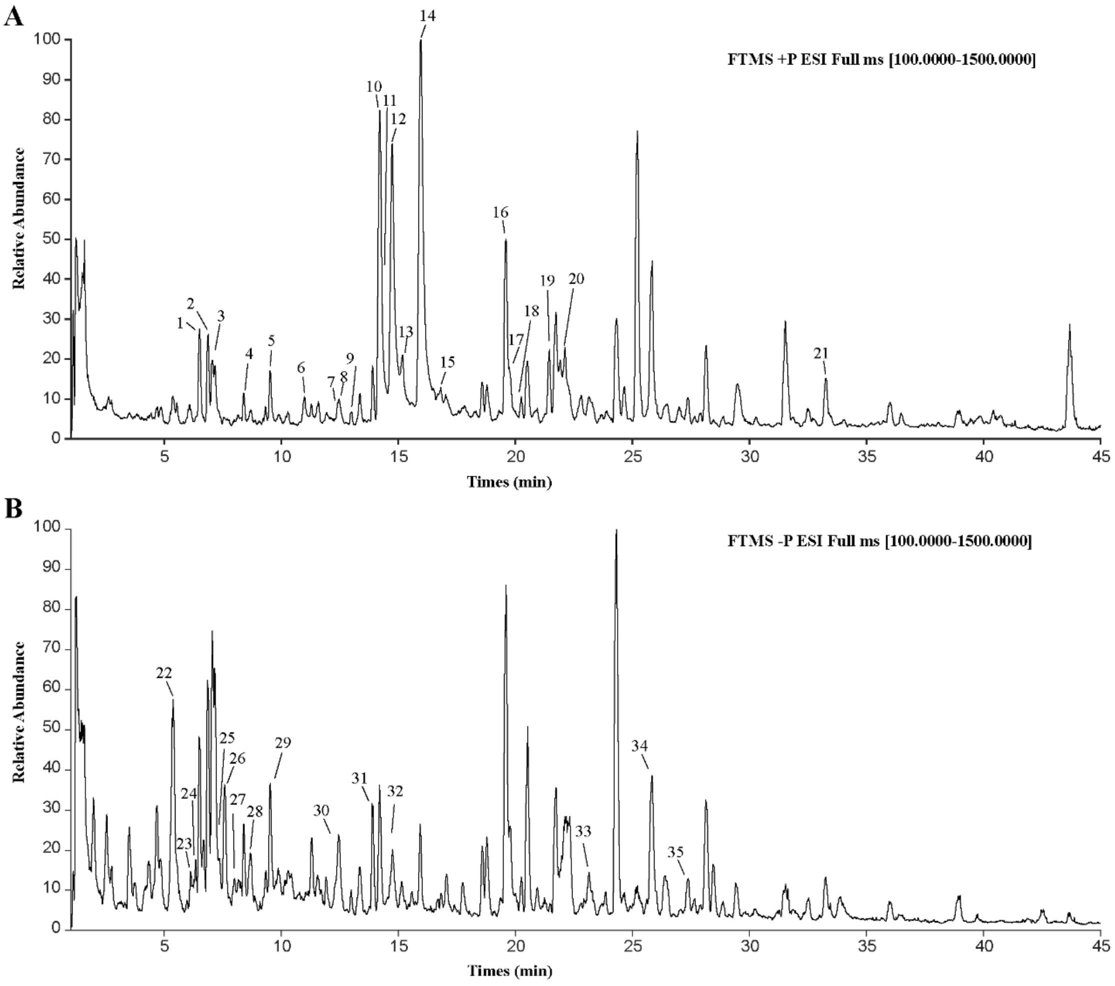

Figure S6. UPLC -MS/MS Analysis of MGFD. (A) Total ion chromatogram of MGFD (positive ions). (B) Total ion chromatogram of MGFD (negative ions). (1) Peoniflorin, (2) Daidzin, (3) Schaftoside, (4) 1,2,3,6-Tetra-O-galloyl-β-D-glucose, (5) Naringenin, (6) Coumarin, (7) Luteolin, (8) Quercetin, (9) Bakuchalcone, (10) Psoralen, (11) 4-Methoxycinnamic acid, (12) Cinnamaldehyde, (13) Psoralidin, (14) Paeonol, (15) Brosimacutin C, (16) Neobavaisoflavone, (17) Poricoic acid C, (18) (+)-Bakuchiol, (19) Protocatechualdehyde, (20) Benzyl cinnamate, (21) Pachymic acid, (22) D(-)-Amygdalin, (23) Catechin, (24) Paeonolide, (25) Vanillin, (26) Oxypaeoniflorin, (27), Gallic acid, (28), Albiflorin, (29) Naringin, (30) Eriodictyol, (31) Benzoylpaeoniflorin, (32) Kaempferol, (33) Bavachin, (34) Isobavachin, (35) Poricoic acid A.
